# Talatisamine, a C_19_-diterpenoid alkaloid from Chinese traditional herbal ‘Chuanwu’

**DOI:** 10.1107/S1600536811044242

**Published:** 2011-11-02

**Authors:** Jun Lei, Ya-Jun Luo, Qing-Quan Bian, Xiong-Qing Wang

**Affiliations:** a Institute of Chemistry and Chemical Engineering, Mianyang Normal University, Mianyang 621000, People’s Republic of China

## Abstract

The title compound [systematic name: (1*S*,4*S*,5*R*,7*S*,8*S*,9*R*,10*R*,11*S*,13*S*,14*S*,16*S*,17*R*)-*N*-methyl-8,14-dihy­droxy-1,16-tri­meth­oxy-4-(meth­oxy­methyl­ene)aconitane], C_24_H_39_NO_5_, was isolated from the roots of *Aconitum carmichaelii* Debx., which is known as ‘Chuanwu’ in Chinese traditional herbal medicine. The mol­ecule has an aconitane carbon skeleton with four six-membered rings and two five-membered rings, including a six-membered N-containing heterocyclic ring. Both five-membered rings adopt envelope conformations. The four six-membered adopt chair conformations. Two intra­molecular O—H⋯O hydrogen bonds occur.

## Related literature

The title compound is an aconitine-type C_19_-diterpenoid alkaloid. For reviews of diterpenoid alkaloids, see: Wang *et al.* (2009 ▶, 2010 ▶). For the chemical structure of the title compound established from NMR and MS data, see: Pelletier *et al.* (1984 ▶). For the total synthesis of the title compound, see: Wiesner *et al.* (1974 ▶). For structures of related C_19_-diterpenoid alkaloids, see: Gao *et al.* (2010 ▶); Tashkhodjaev & Sultankhodjaev (2009 ▶); He *et al.* (2008 ▶). For the absolute configuration of aconitine-type C_19_-diterpenoid alkaloids, see: Pelletier & Djarmati (1976 ▶); Tsuda & Marion (1963 ▶); Zhapova *et al.* (1986 ▶).
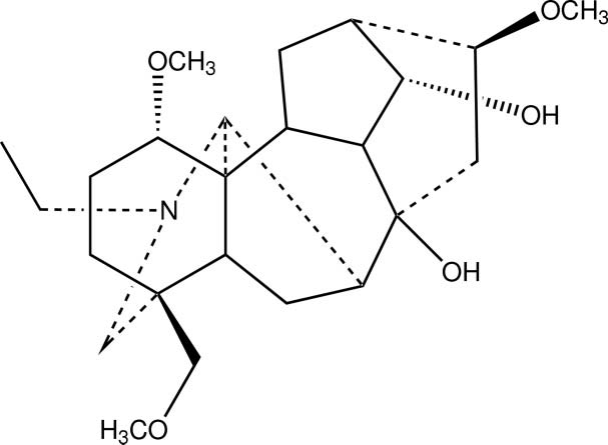


## Experimental

### 

#### Crystal data

C_24_H_39_NO_5_
*M* *_r_* = 421.56
Orthorhombic, 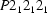

*a* = 9.7124 (4) Å
*b* = 13.9401 (7) Å
*c* = 16.3729 (8) Å
*V* = 2216.77 (17) Å^3^
*Z* = 4
Mo *K*α radiation
μ = 0.09 mm^−1^
*T* = 293 K
0.40 × 0.40 × 0.35 mm

#### Data collection

Oxford Diffraction Xcalibur Eos diffractometer
6644 measured reflections
2572 independent reflections
2068 reflections with *I* > 2σ(*I*)
*R*_int_ = 0.026

#### Refinement

*R*[*F* ^2^ > 2σ(*F* ^2^)] = 0.044
*wR*(*F* ^2^) = 0.097
*S* = 1.04
2572 reflections
277 parameters
H-atom parameters constrained
Δρ_max_ = 0.15 e Å^−3^
Δρ_min_ = −0.15 e Å^−3^

### 

Data collection: *CrysAlis CCD* (Oxford Diffraction, 2009 ▶); cell refinement: *CrysAlis CCD*; data reduction: *CrysAlis RED* (Oxford Diffraction, 2009 ▶); program(s) used to solve structure: *SHELXS97* (Sheldrick, 2008 ▶); program(s) used to refine structure: *SHELXL97* (Sheldrick, 2008 ▶); molecular graphics: *OLEX2* (Dolomanov *et al.*, 2009 ▶); software used to prepare material for publication: *SHELXL97*.

## Supplementary Material

Crystal structure: contains datablock(s) I, global. DOI: 10.1107/S1600536811044242/xu5344sup1.cif

Structure factors: contains datablock(s) I. DOI: 10.1107/S1600536811044242/xu5344Isup2.hkl

Additional supplementary materials:  crystallographic information; 3D view; checkCIF report

## Figures and Tables

**Table 1:** Hydrogen-bond geometry (Å, °)

*D*—H⋯*A*	*D*—H	H⋯*A*	*D*⋯*A*	*D*—H⋯*A*
O2—H2⋯O3	0.82	2.28	2.926 (3)	136
O3—H3⋯O4	0.82	1.95	2.666 (3)	146

**Fig. 1.**
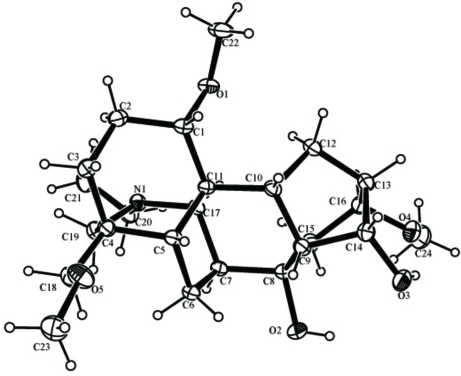
The molecular structure of the title compound with the atom numbering, showing displacement ellipsoids at the 30% probability level.

**Fig. 2.**
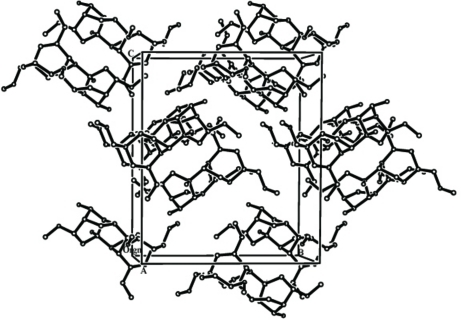
Molecular packing of the title compound.

## supplementary crystallographic information

### Comment

As a famous Chinese traditional herbal, the roots of Aconitum carmichaeli Debx., known as "Chuanwu", has been therapeutically used to the treatment of rheumatic pain, rheumatoid arthritis and some other inflammations. Recently, two important reviews focus on the alkaloids from the genus Aconitum (Wang *et al.*, 2009 and 2010). The title compound, *N*-methyl-8*β*,14*β*-dihydroxy-1*α*,16*β*-trimethoxy-4*β*-(methoxymethylene)aconitane, talatisamine, has been isolated previously from many genus of *Aconitum*L., inculding *A. carmichaeli* Debx. (Pelletier *et al.*, 1984), and its structure was established from the NMR and MS data (Wiesner *et al.*, 1974). However, in our recent investigation, it was isolation from the root of Aconitum carmichaeli Debx. collected in the Jiangyou country, Sichuan Province of China in June, 2011. The crystal structure of talatisamine has not been reported. In view of this, the crystal structure determination of the title compound was carried out and the result is presented here.

The molecular structure of the title compound is shown in Fig. 1. The molecule has a high rigid structure consisting of six main rings (A–F). Six-membered rings A (C1/C2/C3/C4/C5/C11), B (C7/C8/C9/C10/C11/C17) and D(C8/C9/C14/C13/C16/C15) adopt chair conformations; six-membered heterocyclicring E (C4/C5/C11/C17/N1/C19) adopts the same chair conformation. The five-membered rings C (C9/C10/C12/C13/C14) and F (C5/C6/C7/C17/C11) display anenvelope conformation, in which, the C14 and C11 act as the "envelope atom" respectively. Two *cis*-fused ring junctions are observed between rings A/E and between B/C. Two *trans*-fused ring junctions involve rings A/B and also E/F. The crystal structure contains intermolecular O—H···O hydrogen bond between the hydroxyl group and carbonyl O atom. The absolute configuration of the title compound can not be confirmed by the present MoKa diffraction data. But it can be assumed to be the same as that reported for C_19_-diterpenoidalkaloids from the nature (Gao *et al.*, 2010; Tashkhodjaev & Sultankhodjaev, 2009; He *et al.*., 2008; Pelletier & Djarmati, 1976; Tsuda & Marion, 1963; Zhapova *et al.*, 1986).

### Experimental

The title compound was isolated from the roots of Aconitum carmichaeli Debx. according to the literature procedure of Gao *et al.* (2010) and crystals of X-ray quality were grown from MeOH at room temperature by slow evaporation.

### Refinement

Hydroxy H atoms were located in a difference Fourier map and refined as riding in their as-found relative positions with *U*_iso_(H) = 1.5*U*_eq_(O). Other H atoms were located geometrically with C—H = 0.93–0.98 Å, and refined using a riding model with *U*_iso_(H) = 1.5*U*_eq_(C) for methyl and 1.2*U*_eq_(C) for the others. The absolute configuration has not been determined from the X-ray analysis, owing to the absence of strong anomalous scattering.

### Crystal data

**Table:**

C_24_H_39_NO_5_	*F*(000) = 920
*M**_r_* = 421.56	*D*_x_ = 1.263 Mg m^−^^3^
Orthorhombic, *P*2_1_2_1_2_1_	Mo *K*α radiation, λ = 0.7107 Å
Hall symbol: P 2ac 2ab	Cell parameters from 2165 reflections
*a* = 9.7124 (4) Å	θ = 2.9–29.1°
*b* = 13.9401 (7) Å	µ = 0.09 mm^−^^1^
*c* = 16.3729 (8) Å	*T* = 293 K
*V* = 2216.77 (17) Å^3^	Block, colourless
*Z* = 4	0.40 × 0.40 × 0.35 mm

### Data collection

**Table:**

Oxford Diffraction Xcalibur Eos diffractometer	2068 reflections with *I* > 2σ(*I*)
Radiation source: fine-focus sealed tube	*R*_int_ = 0.026
graphite	θ_max_ = 26.4°, θ_min_ = 2.9°
Detector resolution: 16.0874 pixels mm^-1^	*h* = −11→12
ω scans	*k* = −17→16
6644 measured reflections	*l* = −16→20
2572 independent reflections	

### Refinement

**Table:**

Refinement on *F*^2^	Primary atom site location: structure-invariant direct methods
Least-squares matrix: full	Secondary atom site location: difference Fourier map
*R*[*F*^2^ > 2σ(*F*^2^)] = 0.044	Hydrogen site location: inferred from neighbouring sites
*wR*(*F*^2^) = 0.097	H-atom parameters constrained
*S* = 1.04	*w* = 1/[σ^2^(*F*_o_^2^) + (0.0403*P*)^2^ + 0.3082*P*] where *P* = (*F*_o_^2^ + 2*F*_c_^2^)/3
2572 reflections	(Δ/σ)_max_ < 0.001
277 parameters	Δρ_max_ = 0.15 e Å^−^^3^
0 restraints	Δρ_min_ = −0.15 e Å^−^^3^

### Special details

**Table:**

Geometry. All e.s.d.'s (except the e.s.d. in the dihedral angle between two l.s. planes) are estimated using the full covariance matrix. The cell e.s.d.'s are taken into account individually in the estimation of e.s.d.'s in distances, angles and torsion angles; correlations between e.s.d.'s in cell parameters are only used when they are defined by crystal symmetry. An approximate (isotropic) treatment of cell e.s.d.'s is used for estimating e.s.d.'s involving l.s. planes.
Refinement. Refinement of *F*^2^ against ALL reflections. The weighted *R*-factor *wR* and goodness of fit *S* are based on *F*^2^, conventional *R*-factors *R* are based on *F*, with *F* set to zero for negative *F*^2^. The threshold expression of *F*^2^ > σ(*F*^2^) is used only for calculating *R*-factors(gt) *etc*. and is not relevant to the choice of reflections for refinement. *R*-factors based on *F*^2^ are statistically about twice as large as those based on *F*, and *R*- factors based on ALL data will be even larger.

### Fractional atomic coordinates and isotropic or equivalent isotropic displacement parameters (Å^2^)

**Table:**

	*x*	*y*	*z*	*U*_iso_*/*U*_eq_	
O1	0.32334 (17)	0.44018 (14)	0.41854 (12)	0.0435 (5)	
O2	0.89891 (18)	0.25592 (15)	0.37591 (14)	0.0564 (6)	
H2	0.9046	0.2223	0.3350	0.085*	
O3	0.7579 (2)	0.17270 (16)	0.23475 (15)	0.0605 (6)	
H3	0.7429	0.1208	0.2565	0.091*	
O4	0.6335 (2)	0.05223 (14)	0.33745 (13)	0.0561 (6)	
O5	0.7984 (2)	0.70995 (17)	0.35432 (14)	0.0662 (6)	
N1	0.6111 (2)	0.47017 (16)	0.54202 (13)	0.0365 (5)	
C1	0.4310 (2)	0.50466 (19)	0.39450 (17)	0.0368 (6)	
H1	0.4124	0.5232	0.3378	0.044*	
C2	0.4232 (3)	0.5949 (2)	0.44516 (18)	0.0448 (7)	
H2B	0.4167	0.5780	0.5025	0.054*	
H2A	0.3411	0.6306	0.4305	0.054*	
C3	0.5493 (3)	0.6571 (2)	0.43140 (19)	0.0440 (7)	
H3B	0.5462	0.7114	0.4683	0.053*	
H3A	0.5480	0.6816	0.3760	0.053*	
C4	0.6830 (3)	0.60164 (19)	0.44529 (16)	0.0385 (6)	
C5	0.6955 (2)	0.51749 (18)	0.38407 (17)	0.0356 (6)	
H5	0.7034	0.5407	0.3278	0.043*	
C6	0.8211 (3)	0.45461 (19)	0.40829 (18)	0.0402 (7)	
H6A	0.8734	0.4363	0.3603	0.048*	
H6B	0.8812	0.4891	0.4454	0.048*	
C7	0.7596 (2)	0.3657 (2)	0.45023 (17)	0.0375 (6)	
H7	0.8128	0.3489	0.4990	0.045*	
C8	0.7561 (3)	0.2812 (2)	0.38994 (17)	0.0403 (7)	
C9	0.6954 (3)	0.31884 (19)	0.30943 (16)	0.0381 (6)	
H9	0.7625	0.3596	0.2812	0.046*	
C10	0.5624 (2)	0.37418 (19)	0.32477 (16)	0.0356 (6)	
H10	0.5415	0.4092	0.2744	0.043*	
C11	0.5686 (2)	0.44982 (18)	0.39451 (15)	0.0317 (6)	
C12	0.4537 (3)	0.29090 (19)	0.33160 (18)	0.0431 (7)	
H12B	0.4238	0.2833	0.3878	0.052*	
H12A	0.3739	0.3047	0.2980	0.052*	
C13	0.5264 (3)	0.1990 (2)	0.30144 (17)	0.0434 (7)	
H13	0.4657	0.1614	0.2659	0.052*	
C14	0.6491 (3)	0.2379 (2)	0.25413 (17)	0.0459 (7)	
H14	0.6152	0.2659	0.2031	0.055*	
C15	0.6805 (3)	0.1936 (2)	0.42678 (18)	0.0459 (7)	
H15B	0.7498	0.1481	0.4447	0.055*	
H15A	0.6317	0.2152	0.4751	0.055*	
C16	0.5773 (3)	0.13917 (19)	0.37274 (18)	0.0448 (7)	
H16	0.4978	0.1216	0.4064	0.054*	
C17	0.6132 (2)	0.40139 (19)	0.47427 (15)	0.0338 (6)	
H17	0.5530	0.3468	0.4864	0.041*	
C18	0.8026 (3)	0.6714 (2)	0.43440 (19)	0.0491 (8)	
H18B	0.7958	0.7227	0.4742	0.059*	
H18A	0.8892	0.6382	0.4430	0.059*	
C19	0.6885 (3)	0.55955 (19)	0.53215 (17)	0.0425 (7)	
H19A	0.7839	0.5479	0.5465	0.051*	
H19B	0.6525	0.6067	0.5701	0.051*	
C20	0.6367 (3)	0.4256 (2)	0.62167 (17)	0.0474 (7)	
H20B	0.7342	0.4300	0.6341	0.057*	
H20A	0.6127	0.3582	0.6187	0.057*	
C21	0.5563 (4)	0.4716 (2)	0.68945 (19)	0.0646 (9)	
H21A	0.4601	0.4712	0.6759	0.097*	
H21C	0.5868	0.5366	0.6966	0.097*	
H21B	0.5706	0.4365	0.7392	0.097*	
C22	0.1962 (3)	0.4556 (2)	0.3787 (2)	0.0625 (9)	
H22A	0.2084	0.4491	0.3208	0.094*	
H22C	0.1634	0.5189	0.3909	0.094*	
H22B	0.1303	0.4091	0.3973	0.094*	
C23	0.9152 (4)	0.7649 (3)	0.3346 (2)	0.0748 (11)	
H23A	0.9080	0.7870	0.2793	0.112*	
H23B	0.9964	0.7262	0.3404	0.112*	
H23C	0.9210	0.8190	0.3707	0.112*	
C24	0.6424 (4)	−0.0258 (2)	0.3919 (2)	0.0741 (11)	
H24B	0.6771	−0.0810	0.3635	0.111*	
H24A	0.5527	−0.0399	0.4134	0.111*	
H24C	0.7035	−0.0098	0.4359	0.111*	

### Atomic displacement parameters (Å^2^)

**Table:**

	*U*^11^	*U*^22^	*U*^33^	*U*^12^	*U*^13^	*U*^23^
O1	0.0259 (9)	0.0517 (11)	0.0530 (11)	−0.0006 (10)	−0.0017 (8)	0.0075 (10)
O2	0.0364 (11)	0.0614 (14)	0.0715 (15)	0.0142 (11)	−0.0029 (10)	−0.0154 (12)
O3	0.0587 (12)	0.0572 (13)	0.0657 (16)	−0.0016 (12)	0.0165 (11)	−0.0190 (12)
O4	0.0651 (13)	0.0430 (11)	0.0603 (13)	0.0047 (11)	0.0012 (11)	−0.0085 (11)
O5	0.0634 (13)	0.0682 (14)	0.0670 (15)	−0.0224 (13)	−0.0008 (12)	0.0161 (12)
N1	0.0382 (11)	0.0412 (12)	0.0300 (11)	−0.0002 (11)	−0.0029 (10)	−0.0015 (10)
C1	0.0279 (12)	0.0447 (15)	0.0378 (16)	0.0005 (13)	−0.0033 (12)	0.0065 (12)
C2	0.0360 (14)	0.0466 (16)	0.0518 (18)	0.0059 (14)	0.0000 (13)	0.0005 (15)
C3	0.0427 (15)	0.0407 (15)	0.0486 (17)	0.0047 (14)	−0.0016 (13)	−0.0011 (13)
C4	0.0369 (14)	0.0371 (14)	0.0414 (16)	−0.0017 (13)	−0.0041 (12)	−0.0010 (12)
C5	0.0274 (12)	0.0435 (14)	0.0360 (15)	−0.0021 (12)	−0.0004 (11)	0.0008 (13)
C6	0.0276 (12)	0.0482 (16)	0.0449 (16)	−0.0010 (14)	−0.0043 (12)	−0.0064 (14)
C7	0.0285 (12)	0.0430 (15)	0.0410 (16)	0.0022 (12)	−0.0093 (12)	−0.0026 (13)
C8	0.0314 (13)	0.0436 (15)	0.0459 (16)	0.0044 (13)	−0.0037 (12)	−0.0054 (14)
C9	0.0359 (13)	0.0430 (15)	0.0354 (14)	−0.0062 (14)	0.0001 (12)	−0.0016 (12)
C10	0.0316 (12)	0.0445 (14)	0.0305 (14)	−0.0012 (13)	−0.0052 (11)	−0.0007 (12)
C11	0.0255 (11)	0.0377 (14)	0.0318 (14)	0.0004 (12)	−0.0013 (10)	0.0015 (11)
C12	0.0352 (14)	0.0495 (16)	0.0447 (16)	−0.0038 (14)	−0.0087 (13)	−0.0032 (14)
C13	0.0394 (15)	0.0495 (17)	0.0413 (16)	−0.0062 (14)	−0.0032 (13)	−0.0107 (14)
C14	0.0458 (16)	0.0530 (17)	0.0389 (16)	−0.0011 (15)	0.0012 (13)	−0.0081 (14)
C15	0.0501 (17)	0.0405 (15)	0.0471 (17)	0.0035 (15)	−0.0062 (14)	−0.0014 (13)
C16	0.0444 (15)	0.0428 (15)	0.0473 (17)	−0.0024 (14)	0.0063 (14)	−0.0055 (14)
C17	0.0302 (12)	0.0371 (13)	0.0339 (14)	0.0001 (12)	−0.0058 (11)	−0.0021 (12)
C18	0.0472 (16)	0.0451 (16)	0.0550 (19)	−0.0078 (16)	−0.0027 (15)	−0.0015 (15)
C19	0.0428 (15)	0.0443 (15)	0.0406 (15)	−0.0008 (15)	−0.0051 (13)	−0.0056 (13)
C20	0.0462 (15)	0.0544 (17)	0.0416 (16)	0.0001 (15)	−0.0075 (14)	−0.0002 (15)
C21	0.088 (2)	0.068 (2)	0.0375 (17)	0.000 (2)	−0.0023 (17)	−0.0056 (16)
C22	0.0278 (13)	0.071 (2)	0.089 (3)	0.0022 (16)	−0.0100 (16)	0.008 (2)
C23	0.068 (2)	0.073 (2)	0.084 (3)	−0.021 (2)	0.017 (2)	0.006 (2)
C24	0.084 (3)	0.0498 (19)	0.088 (3)	0.009 (2)	−0.001 (2)	−0.003 (2)

### Geometric parameters (Å, °)

**Table:**

O1—C1	1.434 (3)	C9—C10	1.526 (4)
O1—C22	1.413 (3)	C9—C14	1.515 (4)
O2—H2	0.8200	C10—H10	0.9800
O2—C8	1.449 (3)	C10—C11	1.556 (3)
O3—H3	0.8200	C10—C12	1.573 (4)
O3—C14	1.429 (3)	C11—C17	1.532 (3)
O4—C16	1.449 (3)	C12—H12B	0.9700
O4—C24	1.410 (4)	C12—H12A	0.9700
O5—C18	1.418 (4)	C12—C13	1.543 (4)
O5—C23	1.406 (4)	C13—H13	0.9800
N1—C17	1.466 (3)	C13—C14	1.521 (4)
N1—C19	1.464 (3)	C13—C16	1.518 (4)
N1—C20	1.466 (3)	C14—H14	0.9800
C1—H1	0.9800	C15—H15B	0.9700
C1—C2	1.508 (4)	C15—H15A	0.9700
C1—C11	1.540 (3)	C15—C16	1.537 (4)
C2—H2B	0.9700	C16—H16	0.9800
C2—H2A	0.9700	C17—H17	0.9800
C2—C3	1.518 (4)	C18—H18B	0.9700
C3—H3B	0.9700	C18—H18A	0.9700
C3—H3A	0.9700	C19—H19A	0.9700
C3—C4	1.527 (4)	C19—H19B	0.9700
C4—C5	1.548 (4)	C20—H20B	0.9700
C4—C18	1.525 (4)	C20—H20A	0.9700
C4—C19	1.539 (4)	C20—C21	1.501 (4)
C5—H5	0.9800	C21—H21A	0.9600
C5—C6	1.554 (3)	C21—H21C	0.9600
C5—C11	1.561 (3)	C21—H21B	0.9600
C6—H6A	0.9700	C22—H22A	0.9600
C6—H6B	0.9700	C22—H22C	0.9600
C6—C7	1.538 (4)	C22—H22B	0.9600
C7—H7	0.9800	C23—H23A	0.9600
C7—C8	1.537 (4)	C23—H23B	0.9600
C7—C17	1.558 (3)	C23—H23C	0.9600
C8—C9	1.536 (4)	C24—H24B	0.9600
C8—C15	1.548 (4)	C24—H24A	0.9600
C9—H9	0.9800	C24—H24C	0.9600
			
O1—C1—H1	106.9	C8—C15—H15A	107.7
O1—C1—C2	109.6 (2)	C9—C8—C7	107.3 (2)
O1—C1—C11	108.77 (19)	C9—C8—C15	114.9 (2)
O1—C22—H22A	109.5	C9—C10—H10	106.8
O1—C22—H22C	109.5	C9—C10—C11	115.5 (2)
O1—C22—H22B	109.5	C9—C10—C12	101.9 (2)
O2—C8—C7	105.5 (2)	C9—C14—C13	101.2 (2)
O2—C8—C9	108.3 (2)	C9—C14—H14	108.2
O2—C8—C15	108.9 (2)	C10—C9—C8	110.9 (2)
O3—C14—C9	112.7 (2)	C10—C9—H9	110.3
O3—C14—C13	117.8 (2)	C10—C11—C5	111.1 (2)
O3—C14—H14	108.2	C10—C12—H12B	110.4
O4—C16—C13	106.0 (2)	C10—C12—H12A	110.4
O4—C16—C15	113.4 (2)	C11—C1—H1	106.9
O4—C16—H16	108.2	C11—C5—H5	111.4
O4—C24—H24B	109.5	C11—C10—H10	106.8
O4—C24—H24A	109.5	C11—C10—C12	118.3 (2)
O4—C24—H24C	109.5	C11—C17—C7	100.6 (2)
O5—C18—C4	109.1 (2)	C11—C17—H17	110.3
O5—C18—H18B	109.9	C12—C10—H10	106.8
O5—C18—H18A	109.9	C12—C13—H13	111.0
O5—C23—H23A	109.5	H12B—C12—H12A	108.6
O5—C23—H23B	109.5	C13—C12—C10	106.4 (2)
O5—C23—H23C	109.5	C13—C12—H12B	110.4
N1—C17—C7	114.36 (19)	C13—C12—H12A	110.4
N1—C17—C11	110.7 (2)	C13—C14—H14	108.2
N1—C17—H17	110.3	C13—C16—C15	112.6 (2)
N1—C19—C4	114.1 (2)	C13—C16—H16	108.2
N1—C19—H19A	108.7	C14—O3—H3	109.5
N1—C19—H19B	108.7	C14—C9—C8	111.9 (2)
N1—C20—H20B	109.0	C14—C9—H9	110.3
N1—C20—H20A	109.0	C14—C9—C10	102.9 (2)
N1—C20—C21	112.9 (2)	C14—C13—C12	103.1 (2)
C1—C2—H2B	109.5	C14—C13—H13	111.0
C1—C2—H2A	109.5	C15—C16—H16	108.2
C1—C2—C3	110.7 (2)	H15B—C15—H15A	107.1
C1—C11—C5	112.7 (2)	C16—C13—C12	111.0 (2)
C1—C11—C10	107.6 (2)	C16—C13—H13	111.0
C2—C1—H1	106.9	C16—C13—C14	109.4 (2)
C2—C1—C11	117.2 (2)	C16—C15—C8	118.3 (2)
C2—C3—H3B	109.2	C16—C15—H15B	107.7
C2—C3—H3A	109.2	C16—C15—H15A	107.7
C2—C3—C4	112.0 (2)	C17—C7—H7	110.6
H2B—C2—H2A	108.1	C17—C11—C1	117.6 (2)
C3—C2—H2B	109.5	C17—C11—C5	97.86 (19)
C3—C2—H2A	109.5	C17—C11—C10	109.7 (2)
C3—C4—C5	110.7 (2)	C18—C4—C3	107.9 (2)
C3—C4—C19	111.1 (2)	C18—C4—C5	110.3 (2)
H3B—C3—H3A	107.9	C18—C4—C19	108.9 (2)
C4—C3—H3B	109.2	H18B—C18—H18A	108.3
C4—C3—H3A	109.2	C19—N1—C17	117.8 (2)
C4—C5—H5	111.4	C19—N1—C20	111.8 (2)
C4—C5—C6	108.9 (2)	C19—C4—C5	107.9 (2)
C4—C5—C11	109.0 (2)	H19A—C19—H19B	107.6
C4—C18—H18B	109.9	C20—N1—C17	113.2 (2)
C4—C18—H18A	109.9	C20—C21—H21A	109.5
C4—C19—H19A	108.7	C20—C21—H21C	109.5
C4—C19—H19B	108.7	C20—C21—H21B	109.5
C5—C6—H6A	110.7	H20B—C20—H20A	107.8
C5—C6—H6B	110.7	C21—C20—H20B	109.0
C6—C5—H5	111.4	C21—C20—H20A	109.0
C6—C5—C11	104.53 (19)	H21A—C21—H21C	109.5
C6—C7—H7	110.6	H21A—C21—H21B	109.5
C6—C7—C17	102.1 (2)	H21C—C21—H21B	109.5
H6A—C6—H6B	108.8	C22—O1—C1	114.6 (2)
C7—C6—C5	105.31 (19)	H22A—C22—H22C	109.5
C7—C6—H6A	110.7	H22A—C22—H22B	109.5
C7—C6—H6B	110.7	H22C—C22—H22B	109.5
C7—C8—C15	111.4 (2)	C23—O5—C18	113.3 (3)
C7—C17—H17	110.3	H23A—C23—H23B	109.5
C8—O2—H2	109.5	H23A—C23—H23C	109.5
C8—C7—C6	109.8 (2)	H23B—C23—H23C	109.5
C8—C7—H7	110.6	C24—O4—C16	114.6 (2)
C8—C7—C17	112.8 (2)	H24B—C24—H24A	109.5
C8—C9—H9	110.3	H24B—C24—H24C	109.5
C8—C15—H15B	107.7	H24A—C24—H24C	109.5
			
O1—C1—C2—C3	170.4 (2)	C9—C10—C11—C1	174.2 (2)
O1—C1—C11—C5	−170.0 (2)	C9—C10—C11—C5	50.5 (3)
O1—C1—C11—C10	67.2 (2)	C9—C10—C11—C17	−56.6 (3)
O1—C1—C11—C17	−57.3 (3)	C9—C10—C12—C13	−12.0 (3)
O2—C8—C9—C10	−162.8 (2)	C10—C9—C14—O3	−177.0 (2)
O2—C8—C9—C14	82.9 (3)	C10—C9—C14—C13	−50.3 (3)
O2—C8—C15—C16	−108.1 (3)	C10—C11—C17—N1	−176.36 (19)
C1—C2—C3—C4	−53.5 (3)	C10—C11—C17—C7	62.4 (2)
C1—C11—C17—N1	−52.9 (3)	C10—C12—C13—C14	−18.0 (3)
C1—C11—C17—C7	−174.2 (2)	C10—C12—C13—C16	99.0 (2)
C2—C1—C11—C5	−45.0 (3)	C11—C1—C2—C3	45.9 (3)
C2—C1—C11—C10	−167.8 (2)	C11—C5—C6—C7	−13.3 (3)
C2—C1—C11—C17	67.7 (3)	C11—C10—C12—C13	−139.9 (2)
C2—C3—C4—C5	62.1 (3)	C12—C10—C11—C1	−64.6 (3)
C2—C3—C4—C18	−177.1 (2)	C12—C10—C11—C5	171.6 (2)
C2—C3—C4—C19	−57.7 (3)	C12—C10—C11—C17	64.5 (3)
C3—C4—C5—C6	−171.8 (2)	C12—C13—C14—O3	164.7 (2)
C3—C4—C5—C11	−58.4 (3)	C12—C13—C14—C9	41.4 (3)
C3—C4—C18—O5	−59.6 (3)	C12—C13—C16—O4	175.7 (2)
C3—C4—C19—N1	80.8 (3)	C12—C13—C16—C15	−59.8 (3)
C4—C5—C6—C7	103.0 (2)	C14—C9—C10—C11	167.5 (2)
C4—C5—C11—C1	49.3 (3)	C14—C9—C10—C12	37.9 (3)
C4—C5—C11—C10	170.1 (2)	C14—C13—C16—O4	−71.2 (3)
C4—C5—C11—C17	−75.1 (2)	C14—C13—C16—C15	53.3 (3)
C5—C4—C18—O5	61.5 (3)	C15—C8—C9—C10	75.1 (3)
C5—C4—C19—N1	−40.7 (3)	C15—C8—C9—C14	−39.1 (3)
C5—C6—C7—C8	100.3 (2)	C16—C13—C14—O3	46.5 (3)
C5—C6—C7—C17	−19.6 (3)	C16—C13—C14—C9	−76.8 (3)
C5—C11—C17—N1	67.8 (2)	C17—N1—C19—C4	37.6 (3)
C5—C11—C17—C7	−53.4 (2)	C17—N1—C20—C21	−144.9 (2)
C6—C5—C11—C1	165.6 (2)	C17—C7—C8—O2	−178.7 (2)
C6—C5—C11—C10	−73.6 (2)	C17—C7—C8—C9	66.0 (3)
C6—C5—C11—C17	41.1 (2)	C17—C7—C8—C15	−60.6 (3)
C6—C7—C8—O2	68.2 (3)	C18—C4—C5—C6	68.8 (3)
C6—C7—C8—C9	−47.2 (3)	C18—C4—C5—C11	−177.8 (2)
C6—C7—C8—C15	−173.8 (2)	C18—C4—C19—N1	−160.5 (2)
C6—C7—C17—N1	−72.3 (3)	C19—N1—C17—C7	58.9 (3)
C6—C7—C17—C11	46.3 (2)	C19—N1—C17—C11	−53.8 (3)
C7—C8—C9—C10	−49.4 (3)	C19—N1—C20—C21	79.3 (3)
C7—C8—C9—C14	−163.6 (2)	C19—C4—C5—C6	−50.1 (3)
C7—C8—C15—C16	135.9 (2)	C19—C4—C5—C11	63.4 (3)
C8—C7—C17—N1	169.9 (2)	C19—C4—C18—O5	179.7 (2)
C8—C7—C17—C11	−71.5 (3)	C20—N1—C17—C7	−74.2 (3)
C8—C9—C10—C11	47.8 (3)	C20—N1—C17—C11	173.1 (2)
C8—C9—C10—C12	−81.9 (2)	C20—N1—C19—C4	171.3 (2)
C8—C9—C14—O3	−58.0 (3)	C22—O1—C1—C2	86.3 (3)
C8—C9—C14—C13	68.8 (3)	C22—O1—C1—C11	−144.4 (2)
C8—C15—C16—O4	99.8 (3)	C23—O5—C18—C4	−171.7 (2)
C8—C15—C16—C13	−20.5 (3)	C24—O4—C16—C13	−159.0 (3)
C9—C8—C15—C16	13.6 (3)	C24—O4—C16—C15	77.0 (3)

### Hydrogen-bond geometry (Å, °)

**Table:**

*D*—H···*A*	*D*—H	H···*A*	*D*···*A*	*D*—H···*A*
O2—H2···O3	0.82	2.28	2.926 (3)	136.
O3—H3···O4	0.82	1.95	2.666 (3)	146.
